# A Case Report of Drug-Induced Acute Gastric Necrosis

**DOI:** 10.7759/cureus.73696

**Published:** 2024-11-14

**Authors:** Syed Mustafa Haider, Muhammad Nauman Ashraf, Khurram Niaz, Muhammad Hassan Abbas, Usama Rehman

**Affiliations:** 1 Department of General Surgery, Surgical Unit 2, Sheikh Zayed Medical College and Hospital, Rahim Yar Khan, PAK; 2 Section of Histopathology, Department of Pathology, Sheikh Zayed Medical College and Hospital, Rahim Yar Khan, PAK

**Keywords:** acute gastric dilatation, binge eating, exploratory laporotomy, gastric necrosis, venous insufficiency

## Abstract

The stomach has a rich blood supply; for this reason, acute gastric necrosis is a rare clinical condition and needs a high index of suspicion, especially in those patients having no history of an eating disorder and no signs of gastric distension on radiological investigations. We report on a 23-year-old male patient who presented to the emergency department with a one-day history of severe abdominal pain and multiple episodes of vomiting. On examination, his heart rate was 110 beats per minute. He had a non-distended, tense, and tender abdomen, localized to the epigastrium. He gave a history of drug abuse with recreational drugs (heroin, cannabis, and benzodiazepines). During resuscitation, the nasogastric tube yielded an aspirate of about 1 L of dark-colored hemorrhagic fluid. There was no gas under the diaphragm on the erect abdominal X-ray. Six hours post-admission and resuscitation, exploratory laparotomy was performed due to sepsis. During surgery, 250 mL of brownish-red fluid was drained from the peritoneal cavity. Most of the body of the stomach along the greater curvature was gangrenous from the angle of His up to the incisura angularis along the greater curvature. The left gastric and gastroepiploic arteries were found clotted, while the pulsations of other feeding arteries were normal. A sleeve gastrectomy was performed following the resection of the gangrenous portion. Postoperative recovery, initially in the ICU and subsequently in the surgical ward, was uneventful. On follow-up, no weight loss or nutritional deficiency was observed.

## Introduction

The stomach is a highly vascularized organ with a dense intramural vascular anastomotic network [1]. Therefore, acute gastric gangrene is a rare entity due to increased blood flow to the stomach, the first case being reported by Bauman in 1909 [2]. Previous cases have been reported with acute gastric gangrene secondary to gastric dilatation [3] in patients with a history of binge eating or other eating disorders [4]. The known causes are lifestyle habits, underlying morbidities, acute necrotizing inflammation, acute vascular insufficiency, and postoperative complications [5]. Acute gastric necrosis can usually be mistaken for other causes of peritonitis because of its rare occurrence. Thus, they are mostly diagnosed intraoperatively [2]. This case report presents a rare condition of acute gastric necrosis in a young male with no history of binge eating or eating disorder. This case was previously presented as a poster at Surgicon, International Surgical Conference, in Rawalpindi/Islamabad, Pakistan on 11 February 2024.

## Case presentation

**Figure 1:**
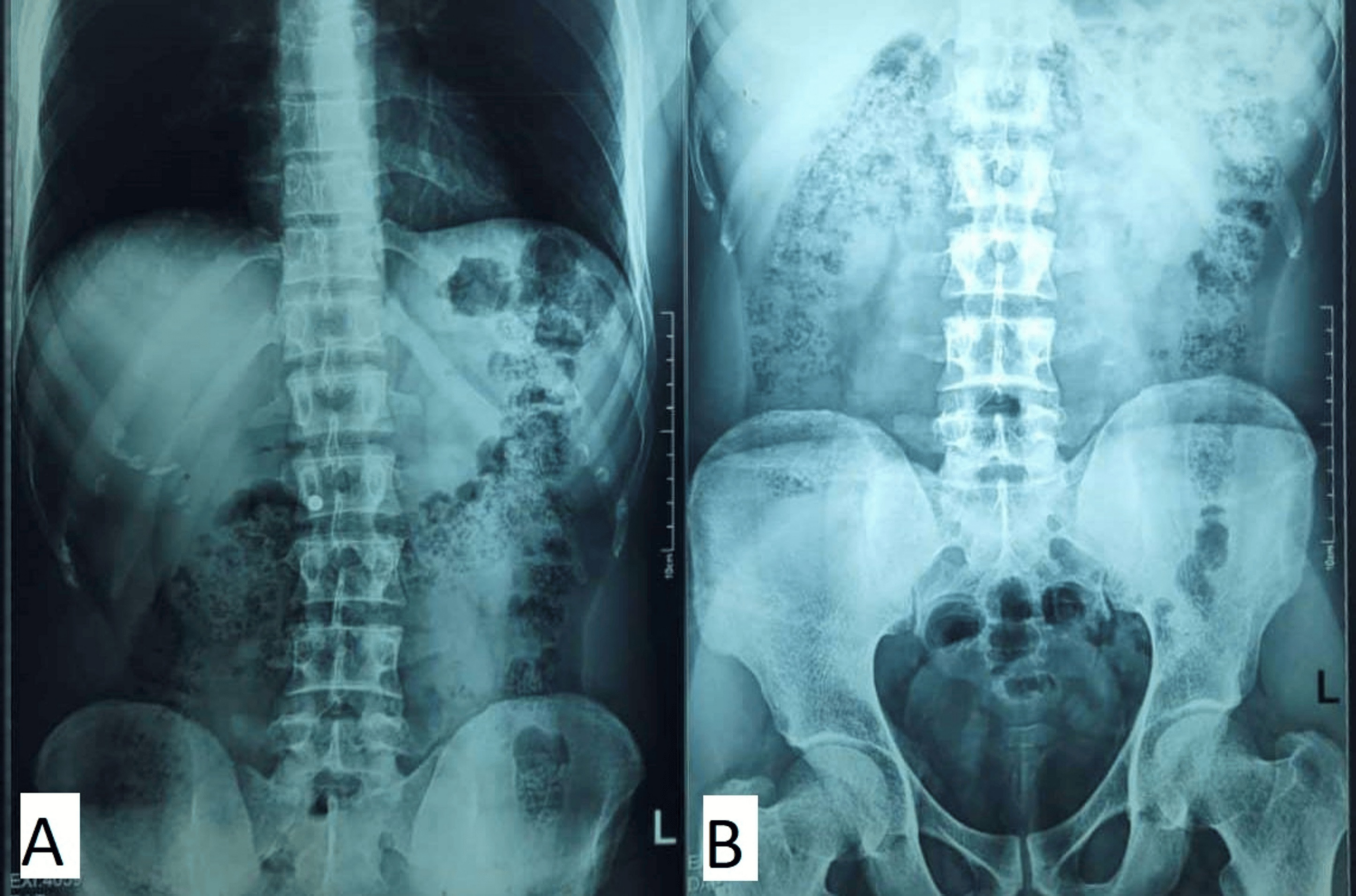
X-ray of the abdomen (PA view) Erect posture shows no sign of visceral perforation or air under the diaphragm (A). There is no sign of gastric distension or intestinal obstruction on a supine view (B). PA, posteroanterior.

A 23-year-old male presented to the emergency department with complaints of severe epigastric pain and vomiting for one day. The pain was of sudden onset, severe in intensity, non-radiating, and generalized. It was followed by 2-3 episodes of vomiting, which contained food particles. On examination, the patient was pale and afebrile, with blood pressure of 100/70 mmHg, pulse rate of 110 beats per minute, and respiratory rate of 22 breaths per minute. The abdomen was tense and tender, more in the epigastrium. Bowel sounds were not present. There was no fluid thrill or shifting dullness. Digital rectal examination was normal. The patient was known to be addicted to opium, benzodiazepines, and cannabis. X-ray of the abdomen supine and erect PA (posteroanterior) view did not show signs of gastric distension and air under the diaphragm (Figure 1). Nasogastric aspirate showed 1 L of dark-colored hemorrhagic fluid. After resuscitation, exploratory laparotomy was performed after 6 h of admission. Peroperatively, 250 mL of brownish-red fluid was drained from the peritoneal cavity. The stomach was found necrosed, along the greater curvature, extending from the angle of His up to the incisura. Left gastric and gastroepiploic arteries were found clotted, while the pulsations of other feeding arteries were normal. A sleeve gastrectomy was performed following resection of the gangrenous portion. Grossly, the stomach was edematous with patchy areas of gangrene over the anterior wall. The postoperative recovery, initially in the ICU and subsequently in the surgical ward, was uneventful.

On follow-up, no weight loss or nutritional deficiency was observed.

**Figure 2:**
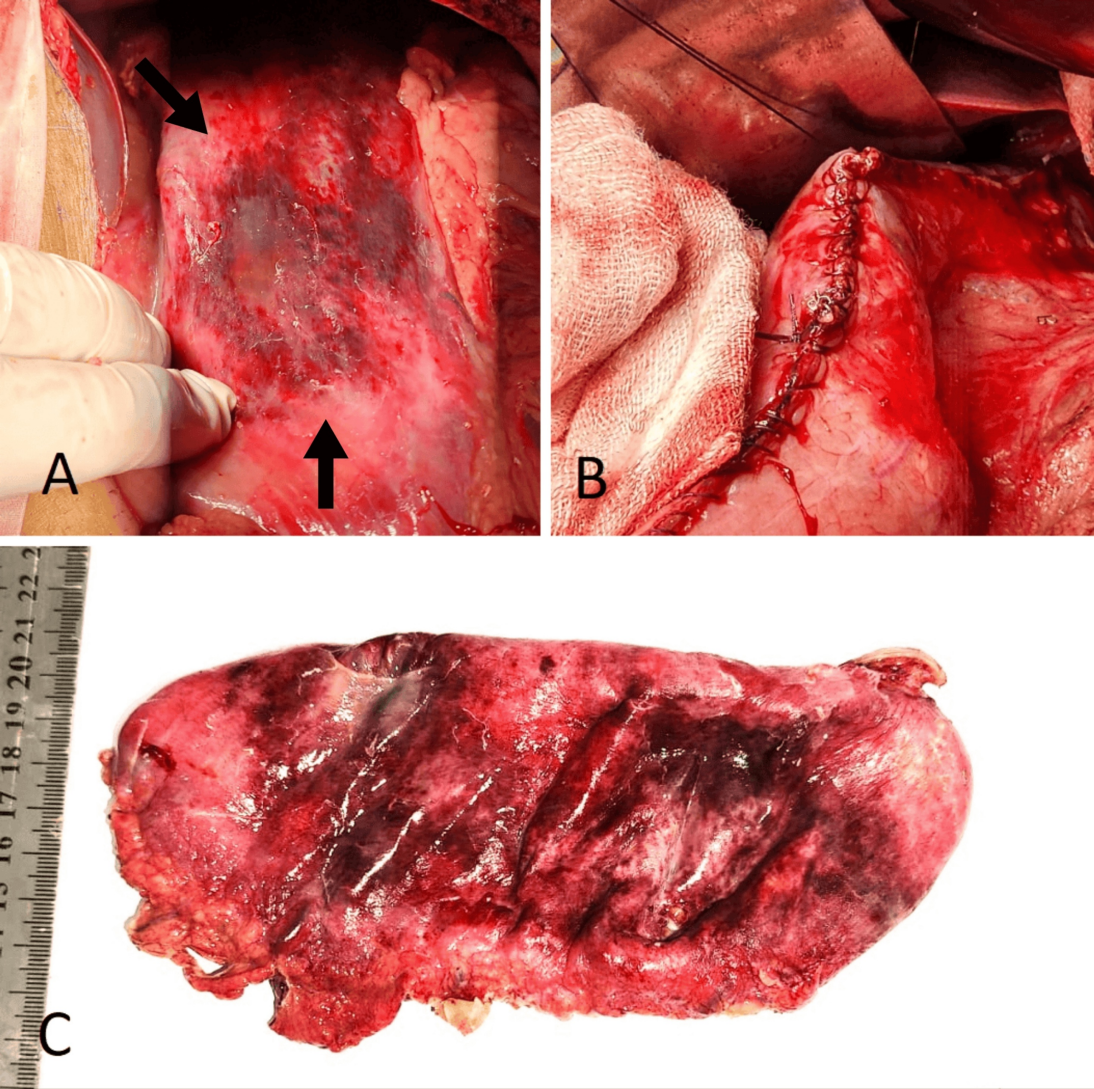
Gross appearance of the stomach An edematous stomach with patchy areas of necrosis of the anterior wall and along the greater curvature is evidenced by the arrows (A). Resection of the gangrenous portion of the stomach, followed by sleeve gastrectomy (B). Gross specimen after excision of the gangrenous portion and sleeve gastrectomy (C).

**Figure 3:**
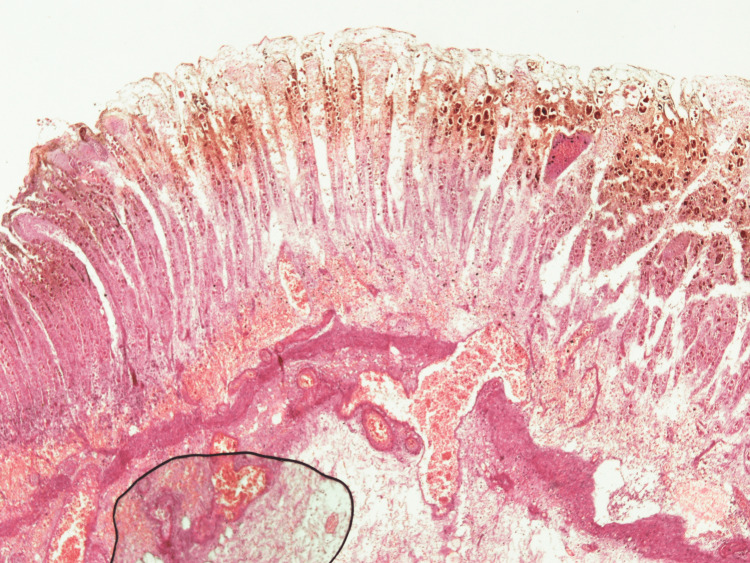
Histology of gastric tissue Full-thickness section of gastric tissue with transmural infarction, necrosis, and many hemosiderin-laden macrophages. Submucosal vessels were dilated and congested, and some were thrombosed. There was an extensive purulent process, extensively destroying the mucosa and submucosa.

Figure 2 shows the gross appearance of the stomach, showing necrosis of the anterior gastric wall and along the greater curvature, evident by dark discoloration. Figure 3 shows the histology of the stomach showing transmural infarction and necrosis.

## Discussion

Acute gastric necrosis is a rare condition due to the abundant blood supply of the stomach [1,4,5]. In experimental models, closure of both the right and left gastric arteries, as well as 80% of the collaterals, is required to produce ischemic necrosis [6]. Frequently described causes are postoperative complications [7], anorexia nervosa and bulimia, psychogenic polyphagia, diabetes mellitus, trauma, electrolyte disturbances, gastric volvulus, and spinal conditions [2,5] secondary to gastric dilatation.

Our case is the first case from Pakistan, reported in the literature. Previously, a few cases of acute gastric gangrene have been reported due to gastric dilatation following bouts of binge eating [2,4,8]. In our case, the patient had a history of abdominal pain and vomiting. There was no history of binge eating, eating disorders, or psychiatric illness.

Yorke et al. reported that in cases of gastric necrosis due to acute gastric dilatation, the intra-gastric pressure of 14 mmHg was sufficient enough to cause venous insufficiency. This, in turn, required at least 3 L of intra-gastric content [2]. In the index case, 1 L of dark-colored hemorrhagic fluid was drained from the nasogastric tube, and there was no sign of gastric dilatation clinically as well as on radiological investigations. Therefore, consideration of gastric necrosis was ignored based on history and clinical assessment. From the history of cannabis, benzodiazepines, and heroin intake, no association has been reported between acute gastric dilatation and the mentioned drug intake. Johnson et al. reported in 2010 the downregulation of striatal D2 receptors in obese rats. This demonstrated that overconsumption of palatable food triggers addiction-like neuroadaptive responses in brain reward circuits and drives the development of compulsive eating. Similar changes are seen with the use of heroin or cocaine, leading to compulsive eating, obesity, and drug addiction [9]. Benzodiazepines are widely used in the treatment of GI illnesses associated with anxiety, with generally no association with other GI side effects except mild sedation and sleepiness [10]. None of the published data suggests any association of the above-mentioned drugs with gastric dilatation leading to necrosis and gangrene.

The unique aspect of our case is that there was no history of eating disorder or binge eating, as well as no evidence of gastric dilatation clinically, radiologically, and peroperatively. Therefore, the diagnosis was made intraoperatively, as mentioned in the previous literature regarding acute gastric necrosis [2]. The affected portion was resected in the form of sleeve gastrectomy. Early diagnosis is very important due to high mortality [2,3]. The treatment options include conservative management if there is no sign of peritonitis, sleeve gastrectomy, total gastrectomy with esophagojejunostomy, or esophagostomy, depending upon the clinical situation. Feeding jejunostomy is preferred to start enteral feeding [3,4].

## Conclusions

Acute gastric necrosis, though rare, should remain a consideration in patients with severe abdominal pain and sepsis, even in the absence of classic risk factors such as eating disorders or gastric distension. Timely surgical intervention, including resection of necrotic gastric tissue, is essential to prevent mortality in such cases. This case emphasizes the importance of maintaining a high degree of clinical suspicion for gastric necrosis in the appropriate context. It highlights the role of early exploratory laparotomy in the absence of laparoscopy in ensuring a favorable outcome.
